# Plasticity of seven-transmembrane-helix receptor heteromers in human vascular smooth muscle cells

**DOI:** 10.1371/journal.pone.0253821

**Published:** 2021-06-24

**Authors:** Lauren J. Albee, Xianlong Gao, Matthias Majetschak

**Affiliations:** 1 Department of Surgery, Burn and Shock Trauma Research Institute, Loyola University Chicago Stritch School of Medicine, Maywood, Illinois, United States of America; 2 Department of Surgery, Morsani College of Medicine, University of South Florida, Tampa, Florida, United States of America; 3 Department of Molecular Pharmacology and Physiology, Morsani College of Medicine, University of South Florida, Tampa, Florida, United States of America; Medical College of Georgia, Augusta, UNITED STATES

## Abstract

Recently, we reported that the chemokine (C-X-C motif) receptor 4 (CXCR4) and atypical chemokine receptor 3 (ACKR3) heteromerize with α_1A/B/D_-adrenoceptors (ARs) and arginine vasopressin receptor 1A (AVPR1A) in recombinant systems and in rodent and human vascular smooth muscle cells (hVSMCs). In these studies, we observed that heteromerization between two receptor partners may depend on the presence and the expression levels of other partnering receptors. To test this hypothesis and to gain initial insight into the formation of these receptor heteromers in native cells, we utilized proximity ligation assays in hVSMCs to visualize receptor-receptor proximity and systematically studied how manipulation of the expression levels of individual protomers affect heteromerization patterns among other interacting receptor partners. We confirmed subtype-specific heteromerization between endogenously expressed α_1A/B/D_-ARs and detected that AVPR1A also heteromerizes with α_1A/B/D_-ARs. siRNA knockdown of CXCR4 and of ACKR3 resulted in a significant re-arrangement of the heteromerization patterns among α_1_-AR subtypes. Similarly, siRNA knockdown of AVPR1A significantly increased heteromerization signals for seven of the ten receptor pairs between CXCR4, ACKR3, and α_1A/B/D_-ARs. Our findings suggest plasticity of seven transmembrane helix (7TM) receptor heteromerization in native cells and could be explained by a supramolecular organization of these receptors within dynamic clusters in the plasma membrane. Because we previously observed that recombinant CXCR4, ACKR3, α_1a_-AR and AVPR1A form hetero-oligomeric complexes composed of 2–4 different protomers, which show signaling properties distinct from individual protomers, re-arrangements of receptor heteromerization patterns in native cells may contribute to the phenomenon of context-dependent GPCR signaling. Furthermore, these findings advise caution in the interpretation of functional consequences after 7TM receptor knockdown in experimental models. Alterations of the heteromerization patterns among other receptor partners may alter physiological and pathological responses, in particular in more complex systems, such as studies on the function of isolated organs or in *in vivo* experiments.

## Introduction

Seven-transmembrane-helix (7TM) receptors, of which the majority are G protein-coupled receptors (GPCRs), play essential roles in many aspects of human physiology and in numerous disease processes. GPCRs are important drug targets, and a large proportion of Federal Drug Administration approved drugs are agonists or antagonists of various GPCRs [1, 2].

While GPCRs were originally thought to function as individual protomers or homodimers, accumulating evidence suggests that many GPCRs can also form heterodimers or hetero-oligomers with other receptor partners, which exhibit pharmacological properties distinct from the individual protomers [3–9].

Chemokine (C-X-C motif) receptor 4 (CXCR4), which is a prototypical GPCR, has been reported to exist as a monomer, dimer, and within nanoclusters comprised of more than three protomers in cells [10, 11]. Moreover, CXCR4 has been shown to form heteromers with multiple other GPCRs, such as chemokine (C-C motif) receptor 2 (CCR2), CCR5, CXCR3, atypical chemokine receptor (ACKR) 3, chemerin receptor 23, β_2_-adrenergic receptor (AR), δ-opioid receptor, cannabinoid receptor 2, protease-activated receptor 1, or the virally-encoded GPCR of Herpesvirus *saimiri*, which is thought to alter the pharmacological properties of the receptor partners [7, 12–21]. We showed previously that CXCR4 and ACKR3 also heteromerize with α_1A/B/D_-ARs and arginine vasopressin receptor 1A (AVPR1A) in recombinant systems and in rodent and human vascular smooth muscle cells (hVSMCs), through which the receptors cross-talk [9, 22–27]. Furthermore, we observed that siRNA knockdown of ACKR3 leads to significant increases of CXCR4:AVPR1A heteromers in the rat aortic smooth muscle cell line A7r5 and in hVSMCs [25], which could point toward interdependency of receptor-receptor interactions in the plasma membrane, i.e. that heteromerization between two receptor partners depends on the presence and the expression levels of other partnering receptors. To test this hypothesis and to gain initial insight into the formation of these receptor heteromers in native cells, we utilized proximity ligation assays (PLA) to visualize receptor-receptor proximity at single molecule resolution [28] and systematically studied how manipulation of the expression levels of individual protomers affect heteromerization patterns among other interacting receptor partners. Although PLA does not provide direct evidence for receptor-receptor interactions, positive signals for protein-protein proximity suggest receptor localization within a distance that is likely to permit direct interactions. The findings of the present study suggest that the heteromerization patterns among CXCR4, ACKR3, α_1A/B/D_-ARs, and AVPR1A are interdependent and demonstrate plasticity of their heteromerization patterns.

## Materials and methods

### Cell culture

hVSMCs (primary aortic smooth muscle cells, ATCC PCS-100-012) were purchased from American Type Culture Collection. As described previously [24, 25], hVSMCs were cultured in vascular basal media (ATCC PCS-100-030) supplemented with the vascular smooth muscle growth kit (ATCC PCS-100-042), containing 100 U/mL penicillin, and 100 μg/mL streptomycin. hVSMCs were used between passages 2–5.

### Proximity Ligation Assays (PLAs)

PLAs were performed as described in detail previously [22–25, 29]. In brief, hVSMCs were grown and fixed on 16-well chamber slides (Nunc). Cells were fixed with 4% (wt/vol) paraformaldehyde for 15 min at room temperature and then blocked overnight at 4°C with 3% (wt/vol) BSA in PBS. To visualize proteins individually, slides were incubated with rabbit anti-AVPR1A (Bioss BS-11598R), mouse anti-ACKR3 (R&D MAB42273), goat anti-CXCR4 (Abcam Ab1670) or rabbit anti-CXCR4 (Alomone Labs ACR-014), mouse anti-α_1A_-AR (Abcam Ab87990) or rabbit anti-α_1A_-AR (Abcam Ab137123), rabbit anti-α_1B_-AR (Abcam Ab169523) or goat anti-α_1B_-AR (Santa Cruz SC27136), and goat anti-α_1D_-AR (Santa Cruz SC27099) at 37°C for 105 min in a humidifying chamber. To visualize receptor–receptor interactions, slides were incubated with a combination of two antibodies raised in differing species as appropriate at 37°C for 105 min in a humidifying chamber. All antibodies were used in dilutions of 1: 500. Slides were then washed with PBS and incubated for 60 min at 37°C in a humidifying chamber with secondary species-specific antibodies conjugated with plus and minus Duolink II PLA probes (1:5), as appropriate. Negative control slides were incubated with omission of one primary antibody and two differing species-specific secondary antibodies. Slides were washed again with PLA wash buffer A (Duolink II) and then incubated with ligation-ligase solution for 30 min at 37°C in a humidifying chamber and also washed with PLA wash buffer A. Finally, slides were incubated with amplification polymerase solution for 100 min at 37°C in a humidifying chamber. Slides were then washed twice with PLA wash buffer B (Duolink II), once with 0.01× PLA wash buffer B and allowed to dry. Slides were then mounted with a minimal volume of Duolink II mounting medium with 4′,6-diamidino-2-phenylindole dihydrochloride (DAPI) overnight, and PLA signals (Duolink In Situ Detection Reagent Red (λexcitation/emission 598/634 nm) were identified as fluorescent spots under a fluorescence microscope (Carl Zeiss Axiovert 200M with EC Plan-Neofluor objective lenses (40 × /1.30 oil) equipped with Axio CamMRc5 (Carl Zeiss) and AxioVision Rel. 4.9.1 (Carl Zeiss) acquisition software) at room temperature. For each vision field 10 z-stack images in 1 μm sections were acquired and compressed. PLA signals were quantified using the Duolink Image Tool software (Sigma-Aldrich). Images were imported in merged.tiff formats containing both signal and nuclei channels. Merged images were visually verified for analytical quality. Comparisons and statistical analyses were performed only when PLA assays were performed on the same day in parallel experiments, and fluorescence microscopy was performed with the identical settings. For each experiment and condition, 10 randomly selected non-overlapping vision fields were analyzed.

### Gene silencing via RNA interference

AVPR1A, CXCR4, and ACKR3 siRNA gene silencing was performed as described previously [22, 24, 25, 30]. In brief, hVSMCs were grown in 2 ml Accell siRNA delivery media per well (Dharmacon) in six-well plates (Nunc). Commercially available Accell AVPR1A, CXCR4, or ACKR3 siRNA was reconstituted with 1× siRNA buffer to a stock concentration of 100 μM. Cells were then transfected with 1 μM siRNA and incubated for 72 h at 37°C, 5% CO2. Accell NT-siRNA pool was used as a negative control. After 72 h, cells were assayed for receptor cell surface expression

### Data analyses

Data are expressed as mean ± standard error. Student’s t-test or One-way analyses of variance (ANOVA) with Dunnett’s multiple comparison post hoc test for multiple comparisons were used to assess statistical significance, as appropriate. A two-tailed p<0.05 was considered significant. All analyses were calculated with the GraphPad Prism 8, Version 8.4.0 software.

## Results and discussion

### Heteromerization between AVPR1A and α_1_-AR subtypes

**Fig 1:**
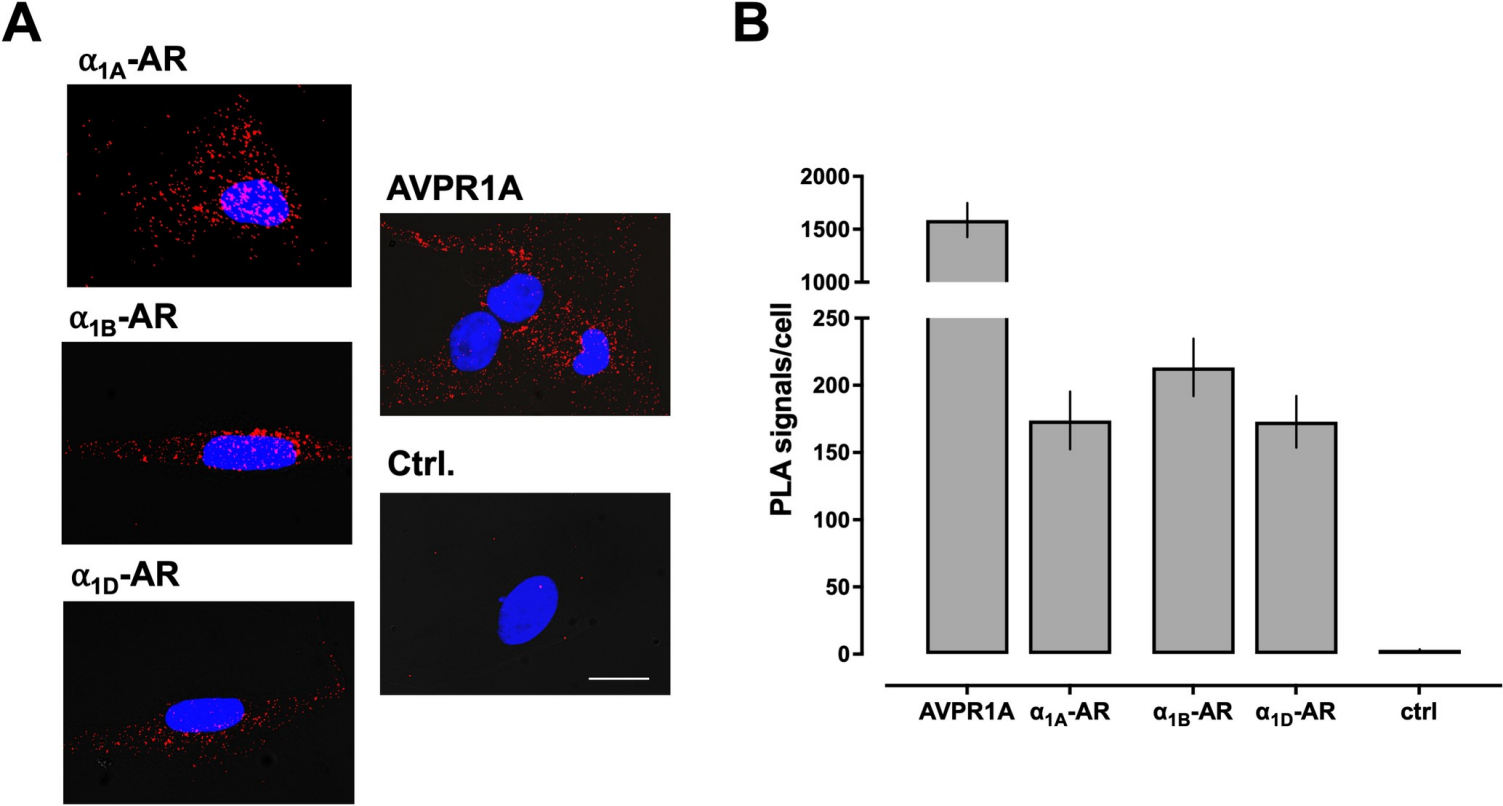
Detection of a_1A/B/D_-AR and AVPR1A in hVSMCs. (**A**) Representative PLA images for the detection of individual receptor protomers. Images show merged PLA/4′,6-diamidino-2-phenylindole dihydrochloride (DAPI) signals. Ctrl: Omission of primary antibody. Scale bar = 10 μm. (**B**) Quantification of PLA signals from n = 4 independent experiments with n = 10 images per experiment.

**Fig 2:**
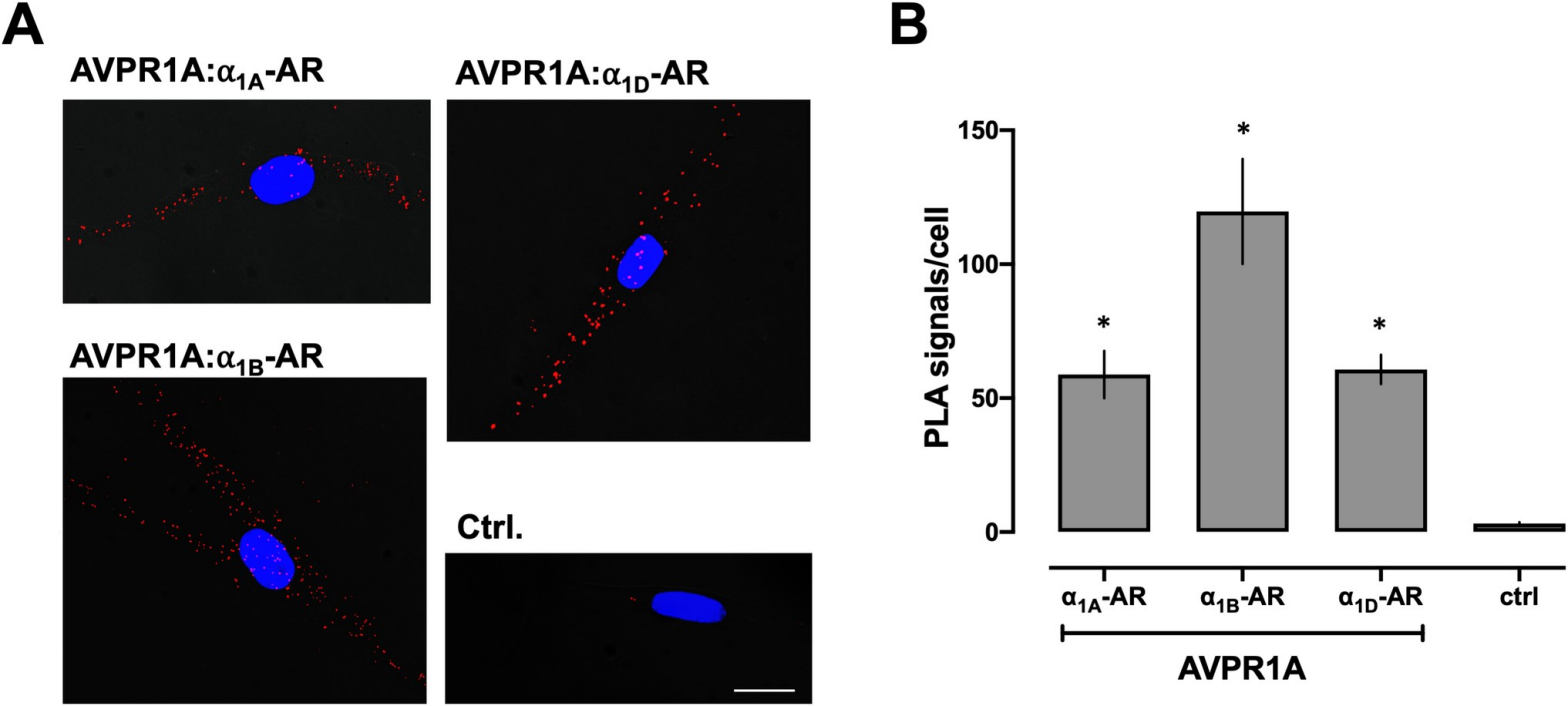
AVPR1A heteromerizes with a_1A/B/D_-ARs in hVSMCs. (**A**) Representative PLA images for the detection of receptor–receptor interactions. Images show merged PLA/4′,6-diamidino-2-phenylindole dihydrochloride (DAPI) signals. Ctrl: Omission of one primary antibody. Scale bar = 10 μm. (**B**) Quantification of PLA signals from n = 4 independent experiments with n = 10 images per experiment. *: p<0.05 vs. ctrl.

We showed previously that CXCR4 and ACKR3 heteromerize with α_1A/B/D_-ARs and AVPR1A in recombinant systems and in hVSMCs [22–26]. Furthermore, we previously observed that recombinant AVPR1A interacts with all recombinant α_1_-AR subtypes, as assessed by intermolecular bioluminescence energy transfer assays in HEK293T cells [9]. Because heteromerization between endogenously expressed AVPR1A and α_1A/B/D_-ARs has not been reported, we first tested whether AVPR1A heteromerizes with any of the α_1_-AR subtypes in hVSMCs. Consistent with our previous findings [22, 24, 25], AVPR1A and all α_1_-AR subtypes could be visualized in hVSMCs when PLA was performed to detect each protomer individually (Fig 1A and 1B). When PLA was performed to detect protein-protein interactions, we observed that AVPR1A constitutively heteromerizes with α_1A_-AR, α_1B_-AR, and α_1D_-AR (Fig 2A and 2B), which confirms our previous findings in a recombinant system.

**Fig 3:**
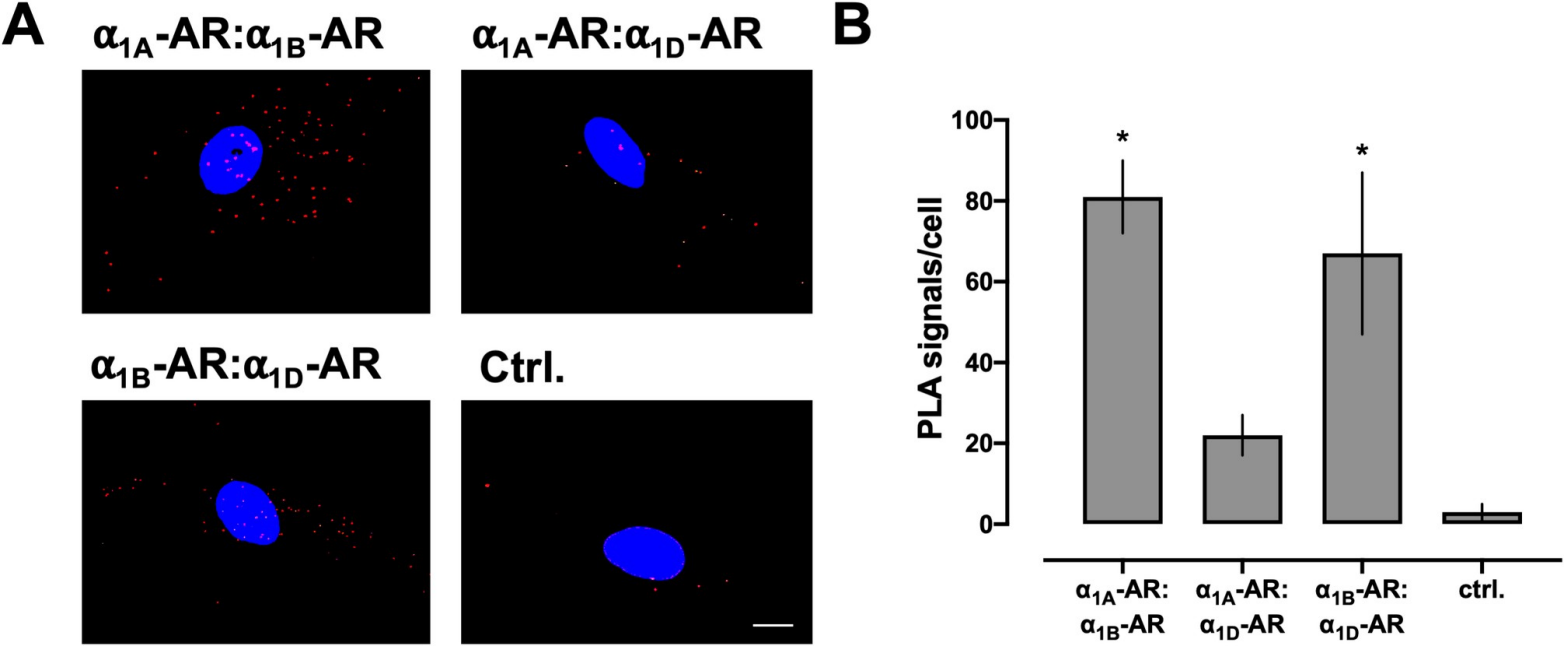
α_1_-ARs form heteromeric complexes in hVSMCs. (**A**) Representative PLA images for the detection of receptor–receptor interactions. Images show merged PLA/4′,6-diamidino-2-phenylindole dihydrochloride (DAPI) signals. Ctrl: Omission of one primary antibody. Scale bar = 10 μm. (**B**) Quantification of PLA signals from n = 3 independent experiments with n = 10 images per experiment. *: p<0.05 vs. ctrl.

Homodimerization of α_1a/b/d_-ARs and heterodimerization of α_1b_-AR with α_1a_-AR and α_1d_-AR has previously been observed in co-immunoprecipitation experiments with epitope-tagged recombinant receptors [31]. To confirm these observations in native cells, we screened for interactions between α_1A/B/D_-ARs. As shown in Fig 3A and 3B, we observed positive PLA signals for proximity between α_1A_-AR and α_1B_-AR, and for proximity between α_1B_-AR and α_1D_-AR (p<0.01 vs. ctrl. for both). PLA signals for proximity between α_1A_-AR and α_1D_-AR were not significantly different from negative control PLA signals (p = 0.17), which is in agreement with previous findings in a recombinant system [31].

### Re-organization of heteromers between α_1A/B/D_-ARs upon depletion of CXCR4 and ACKR3

**Fig 4:**
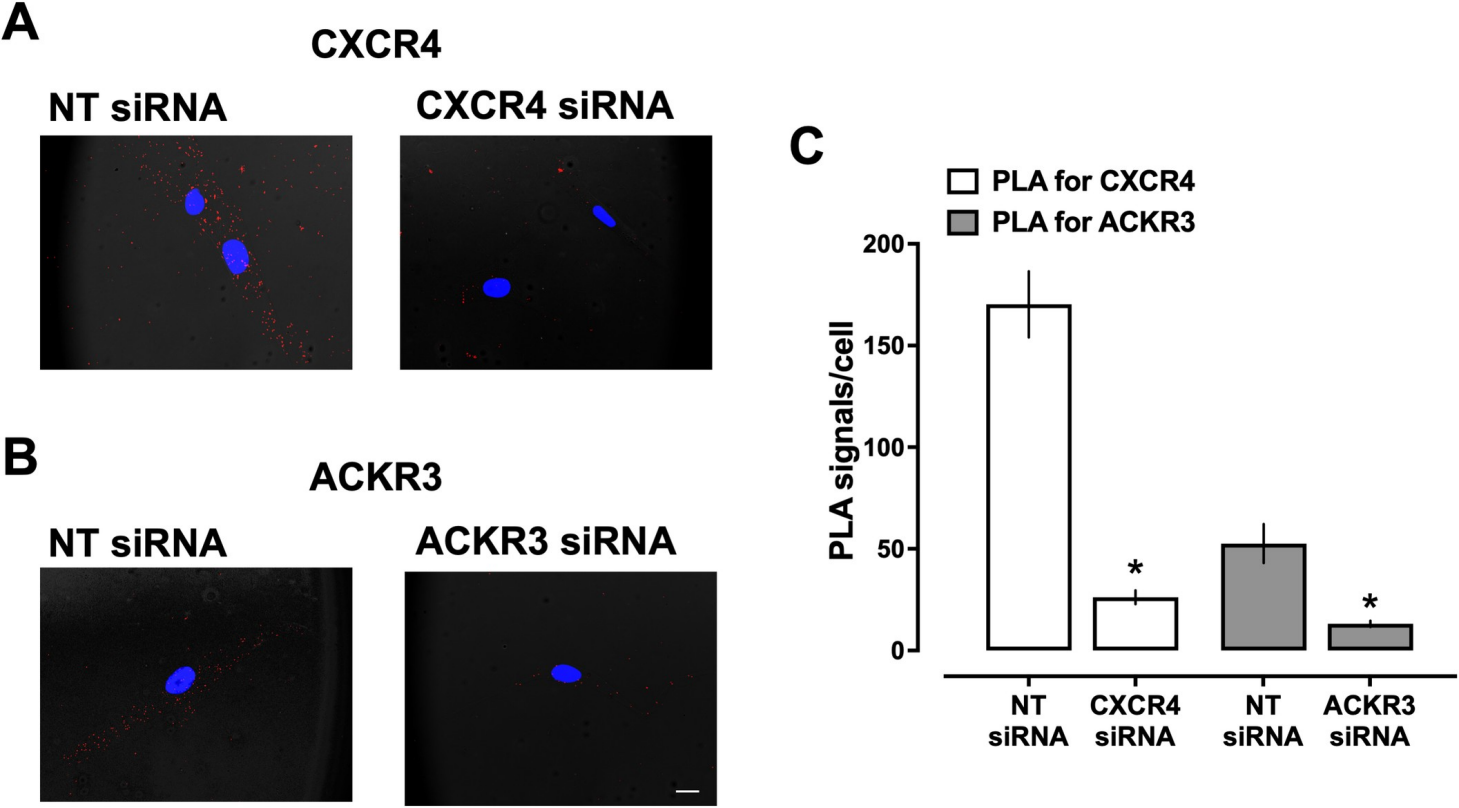
siRNA knockdown of CXCR4 and ACKR3 in hVSMCs. (**A**) Representative PLA images for the detection of CXCR4 in hVSMC after incubation with non-targeting (NT) or CXCR4 siRNA. Images show merged PLA/4′,6-diamidino-2-phenylindole dihydrochloride (DAPI) signals. (**B**) Representative PLA images for the detection of ACKR3 in hVSMC after incubation with NT or ACKR3 siRNA. Images show merged PLA/4′,6-diamidino-2-phenylindole dihydrochloride (DAPI) signals. Scale bar = 10 μm. (**C**) Quantification of PLA signals for the detection of CXCR4 and ACKR3 after incubation with siRNA, as in A/B. N = 3 independent experiments with n = 10 images per experiment. *: p<0.05 vs. cells incubated with NT siRNA.

Because CXCR4 and ACKR3 heteromerize with α_1A/B/D_-ARs in hVSMCs, we tested whether depletion of CXCR4 or ACKR3 from the cell surface by gene silencing via RNA interference influences the heteromerization patterns between α_1A/B/D_-ARs. We showed previously that siRNA knockdown of CXCR4 or ACKR3 does not influence cell surface expression levels of α_1A/B/D_-AR in hVSMCs [22, 24]. As compared with cells incubated with non-targeting (NT)-siRNA, PLA signals for CXCR4 were reduced by 85% after incubation with CXCR4-siRNA (PLA signals/cell: NT-siRNA– 170 ± 16; CXCR4-siRNA: 26 ± 3; p<0.01), and for ACKR3 by 75% after incubation with ACKR3-siRNA (PLA signals/cell: NT-siRNA– 53 ± 10; ACKR3-siRNA: 13 ± 1.5; p<0.01), respectively (Fig 4A–4C).

**Fig 5:**
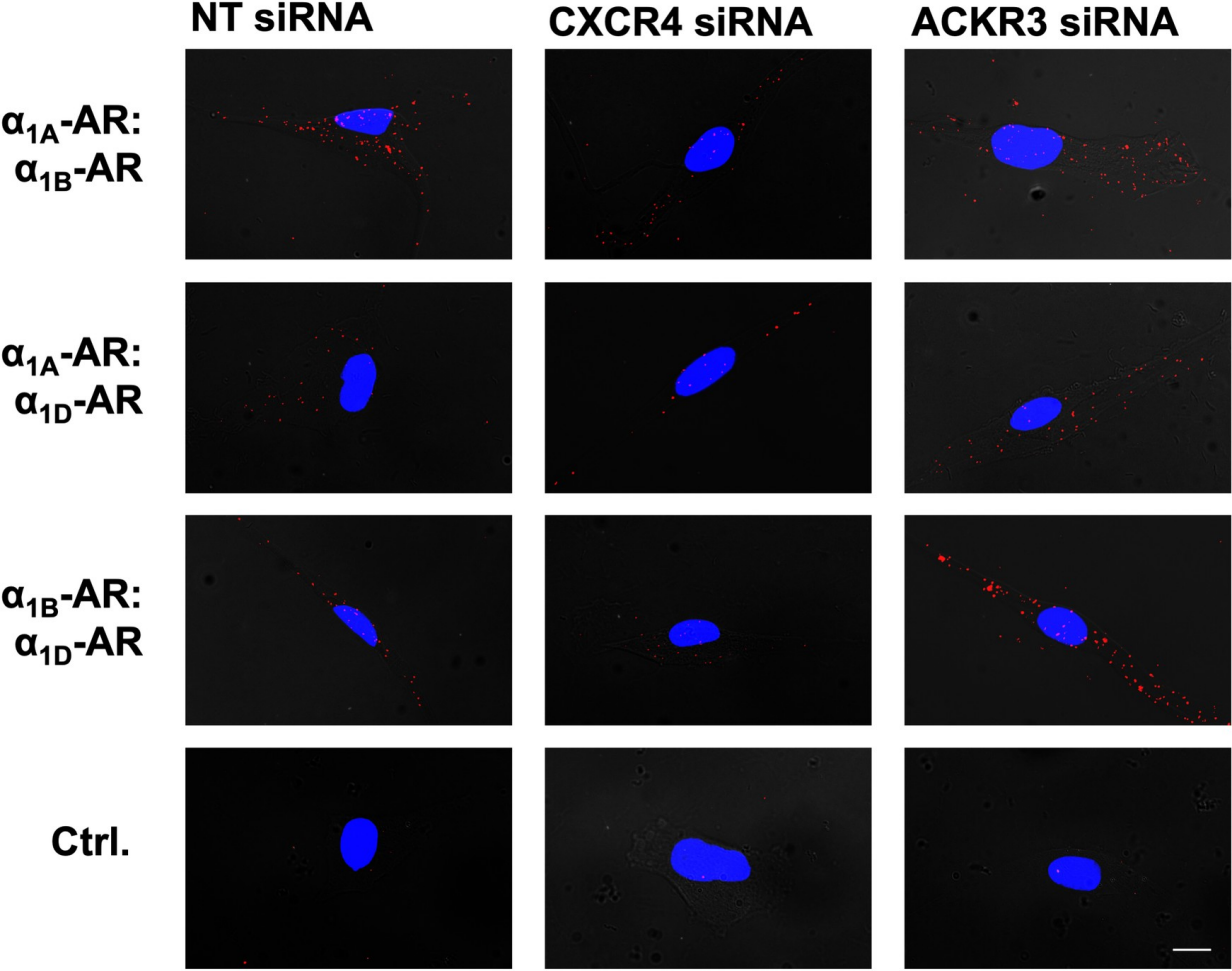
Detection of α_1A/B/D_-AR heteromers after depletion of CXCR4 or ACKR3 in hVSMCs. Representative PLA images for the detection of α_1A/B/D_-AR heteromers in hVSMCs after incubation with non-targeting (NT) siRNA (left), CXCR4 siRNA (center), or ACKR3 siRNA (right). Ctrl: Omission of one primary antibody. Images show merged PLA/4′,6-diamidino-2-phenylindole dihydrochloride (DAPI) signals. Scale bar = 10 μm.

**Fig 6:**
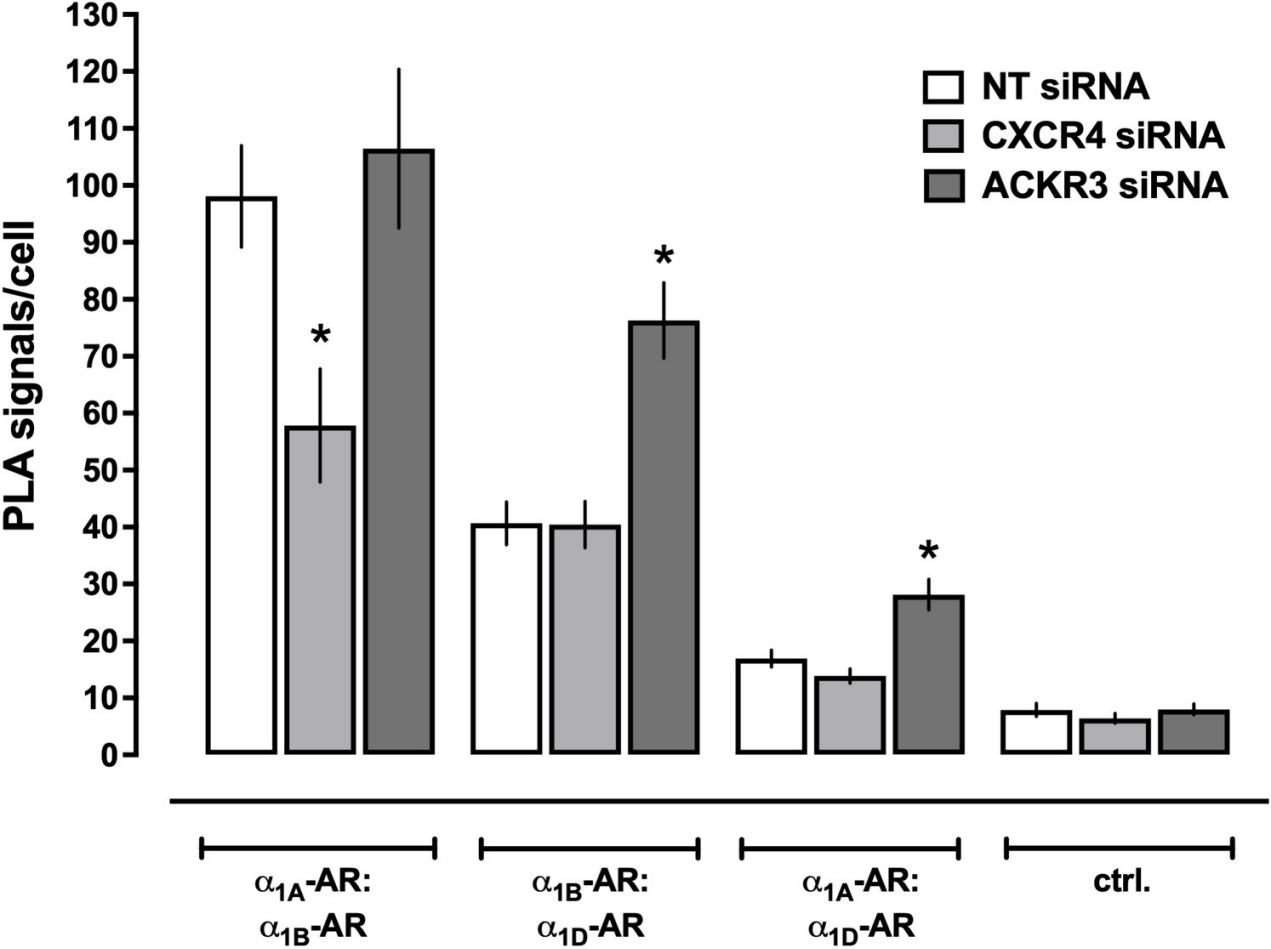
Re-organization of α_1A/B/D_-AR heteromers after depletion of CXCR4 or ACKR3 in hVSMCs. Quantification of PLA signals for the detection of receptor-receptor interactions in hVSMCs after incubation with non-targeting (NT) siRNA, CXCR4 siRNA, or ACKR3 siRNA, as in Fig 5. N = 3 independent experiments with n = 10 images per experiment. *: p<0.05 vs. cells incubated with NT siRNA.

We observed that depletion of CXCR4 from the cell surface significantly reduced PLA signals corresponding to α_1A_-AR:α_1B_-AR heteromers, but did not affect PLA signals for α_1B_-AR:α_1D_-AR or α_1A_-AR:α_1D_-AR heteromers (Figs 5 and 6). In contrast, depletion of ACKR3 from the cell surface did not affect PLA signals for α_1A_-AR:α_1B_-AR heteromers, but significantly increased PLA signals for α_1B_-AR:α_1D_-AR and α_1A_-AR:α_1D_-AR heteromers (Figs 5 and 6). These observations suggest that ACKR3 and α_1D_-AR compete for heteromerization interfaces with α_1A_-AR and α_1B_-AR, which are occupied by α_1D_-AR upon depletion of ACKR3. The finding that CXCR4 knockdown reduces α_1A_-AR:α_1B_-AR heteromers, however, could be explained by a higher-order receptor complex, in which CXCR4 facilitates proximity between α_1A_-AR and α_1B_-AR.

### Re-organization of heteromers between α_1A/B/D_-ARs, CXCR4, and ACKR3 upon depletion of AVPR1A in hVSMCs

**Fig 7:**
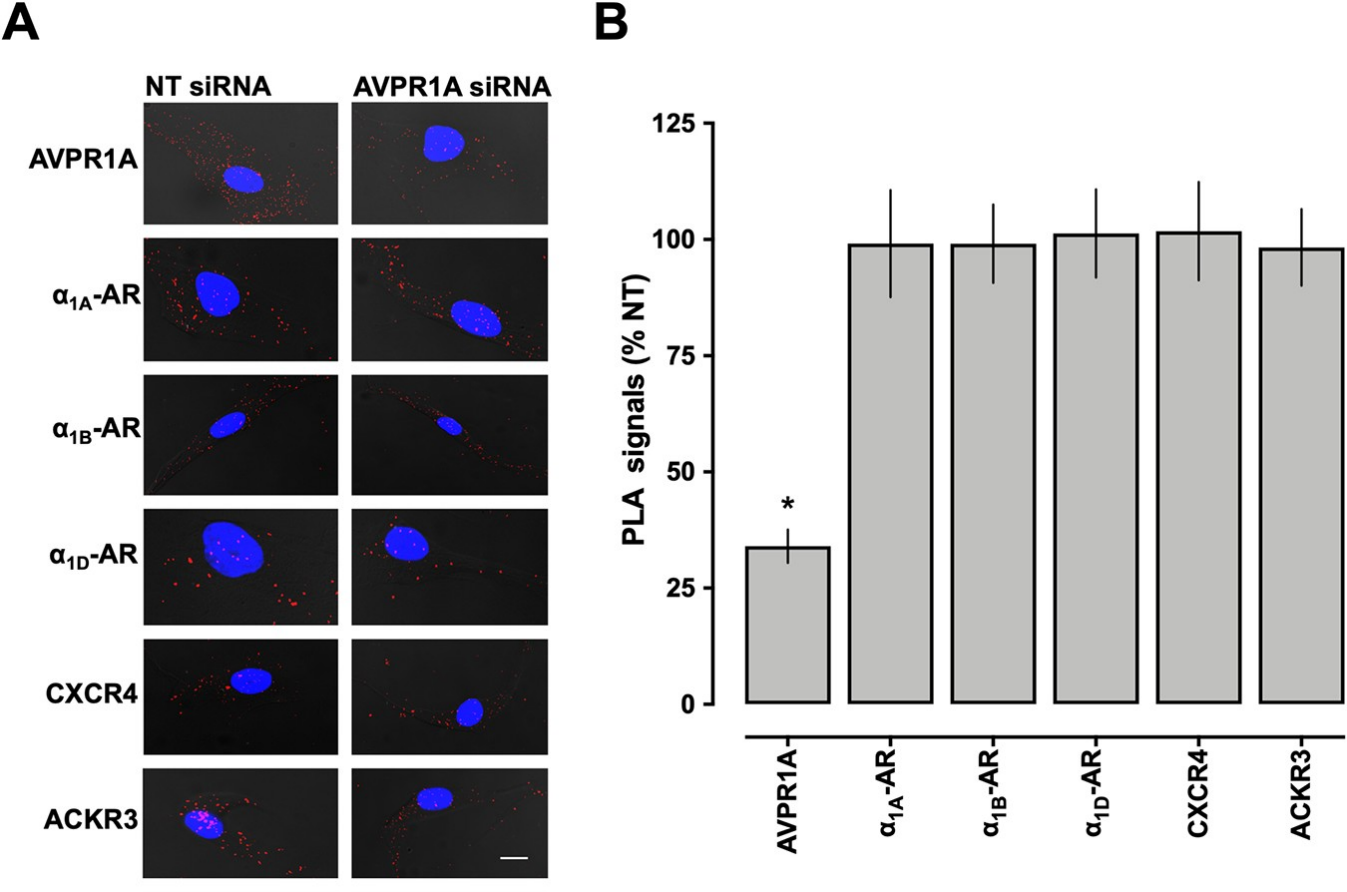
siRNA knockdown of AVPR1A in hVSMCs. (**A**) Representative PLA images for the detection of individual receptor protomers in hVSMC after incubation with non-targeting (NT) siRNA or AVPR1A siRNA. Images show merged PLA/4′,6-diamidino-2-phenylindole dihydrochloride (DAPI) signals. Scale bar = 10 μm. (**B**) Quantification of PLA signals for the detection of individual receptor protomers in hVSMCs after incubation with non-targeting (NT) siRNA or AVPR1A siRNA, as in A. PLA signals in cells incubated with AVPR1A siRNA are expressed as % of PLA signals in cells incubated with NT siRNA (= 100%). n = 4 independent experiments with n = 10 images per condition and experiment. *: p<0.05 vs. cells incubated with NT siRNA.

Because our previous studies, in combination with the findings of the present study, suggest that AVPR1A heteromerizes with each of the CXCR4, ACKR3, and α_1A/B/D_-AR protomers in hVSMCs, we tested whether depletion of AVPR1A from the cell surface would also affect heteromerization patterns between all other receptor pairs. As compared with hVSMCs incubated with NT siRNA, PLA signals for AVPR1A in hVSMCs after incubation with AVPR1A siRNA were reduced by 66±4% (Fig 7A and 7B). PLA signals for ACKR3, CXCR4, and α_1A/B/D_-ARs were indistinguishable in hVSMCs incubated with AVPR1A-siRNA and NT-siRNA (Fig 7A and 7B).

**Fig 8:**
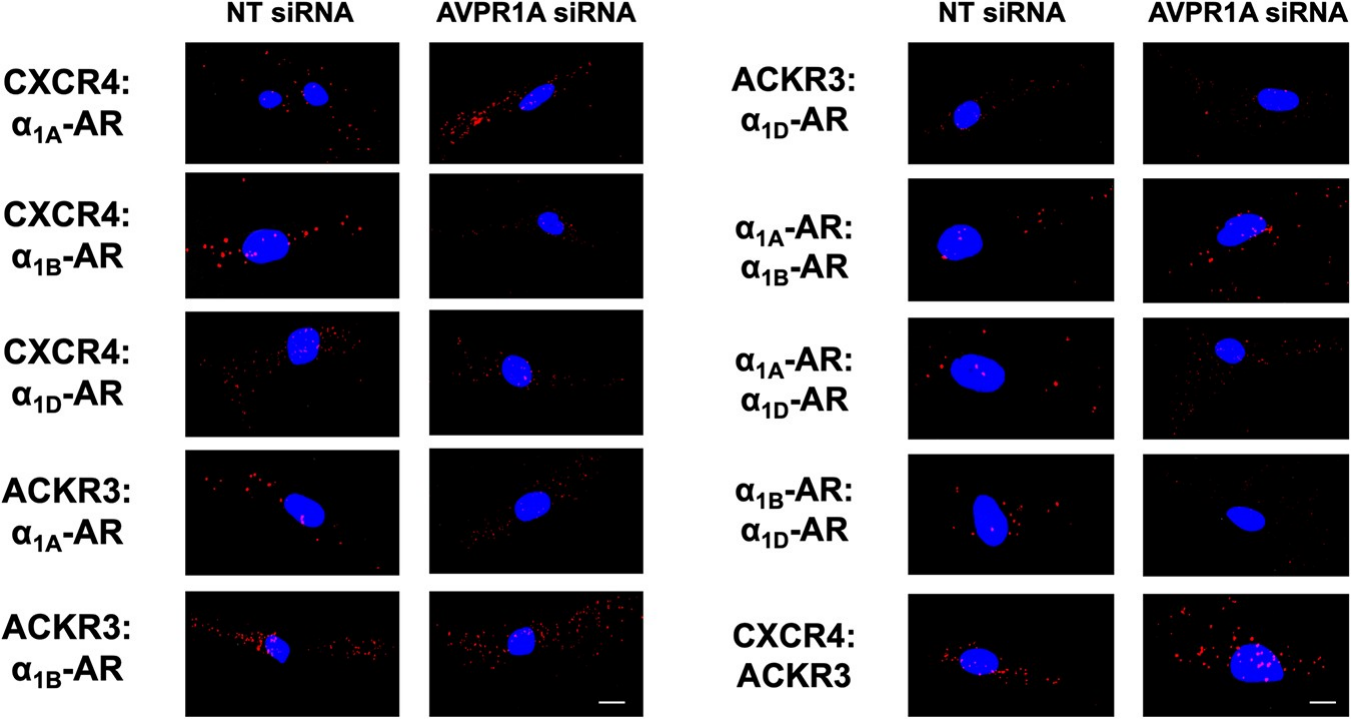
Detection of heteromers between α_1A/B/D_-ARs, CXCR4, and ACKR3 upon depletion of AVPR1A in hVSMCs. Representative PLA images for the detection of receptor-receptor interactions in hVSMC after incubation with non-targeting (NT) siRNA or AVPR1A siRNA. Images show merged PLA/4′,6-diamidino-2-phenylindole dihydrochloride (DAPI) signals. Scale bar = 10 μm.

**Fig 9:**
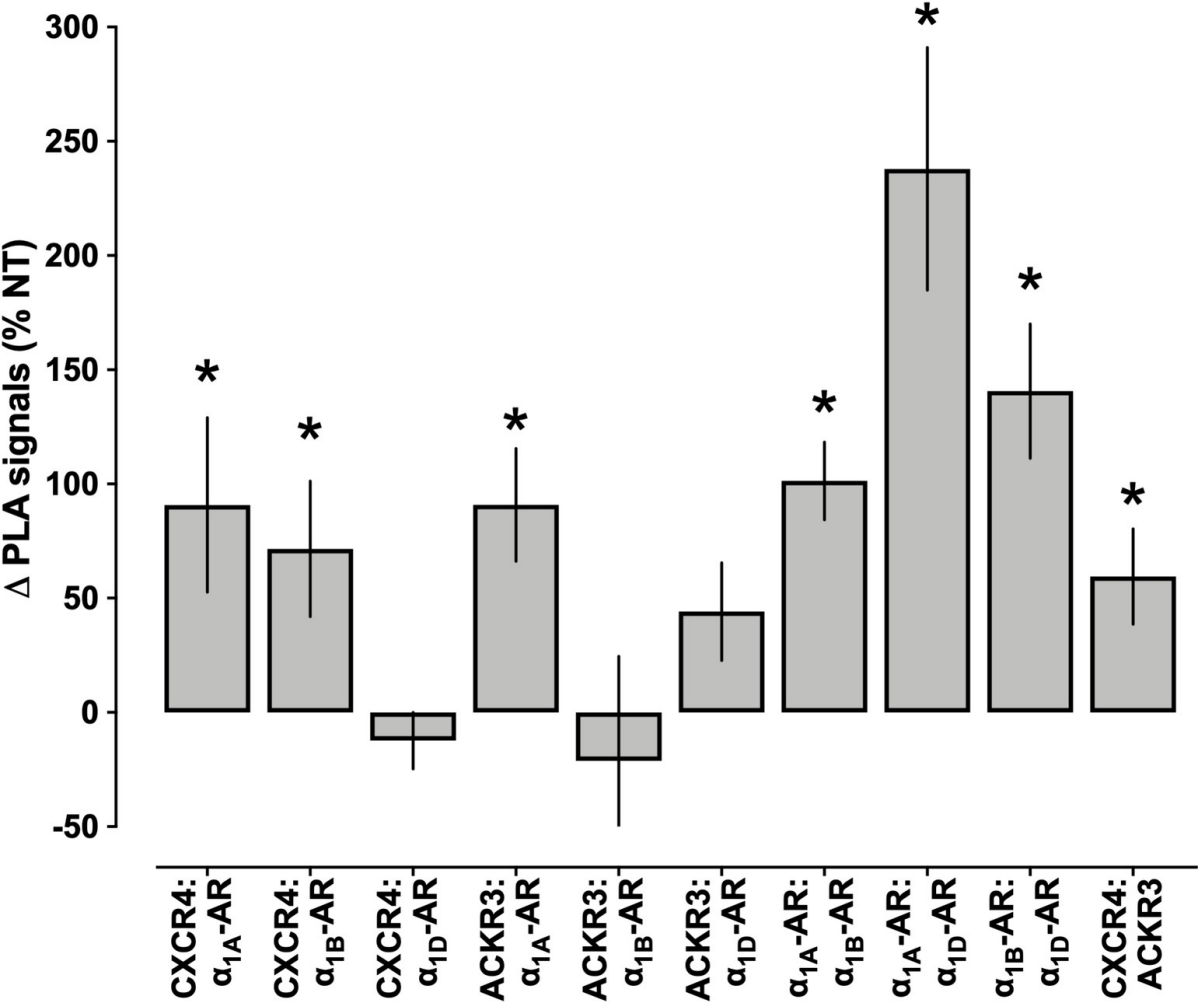
Re-organization of heteromers between α_1A/B/D_-ARs, CXCR4, and ACKR3 upon depletion of AVPR1A in hVSMCs. Quantification of PLA signals for the detection of receptor-receptor interactions in hVSMCs after incubation with non-targeting (NT) siRNA or AVPR1A siRNA, as in Fig 8. N = 4 independent experiments with n = 10 images per experiment. ΔPLA signals (%NT): Change in PLA signals in cells incubated with AVPR1A siRNA in % of PLA signals in cells incubated with NT siRNA (= 100%). *: p<0.05 vs. cells incubated with NT siRNA.

Quantification of the PLA signals for all combinations of heteromeric complexes between ACKR3, CXCR4, and α_1A/B/D_-ARs showed significant increases after incubation with AVPR1A siRNA for the following receptor pairs, as compared with hVSMCs incubated with NT-siRNA (Figs 8 and 9): CXCR4:α_1A_-AR– 91±38%; CXCR4:α_1B_-AR– 72±30%; ACKR3:α_1A_-AR– 91±25%; α_1A_-AR:α_1B_-AR– 101±17%; α_1A_-AR:α_1D_-AR– 238±53%; α_1B_-AR:α_1D_-AR– 141±29%; CXCR4:ACKR3–59±21%. PLA signals suggestive of heteromerization between CXCR4 and α_1D_-AR and between ACKR3 and α_1B/D_-ARs were not significantly affected by AVPR1A knockdown.

Our observations further support the concept that heteromeric receptor complexes exist in a dynamic equilibrium on the cell surface, in which depletion of one receptor heteromerization partner leads to the re-arrangement of multiple heteromeric receptor complexes among other partnering receptors [24, 25]. Such a behavior is in agreement with previous studies of recombinant β_2_-AR and CXCR4, which were found to exist in a dynamic equilibrium of monomers, dimers, and higher-order homomers in the plasma membrane [10, 32] and with our previous findings that peptides derived from transmembrane domains of ACKR3 interfere with CXCR4:ACKR3 heteromerization but increase ACKR3:α_1A_-AR, CXCR4:α_1A_-AR, and CXCR4:α_1B_-AR heteromers [24]. The finding that changes in PLA signals for the various heteromers after AVPR1A knockdown differed largely among the various receptor pairs could be attributed to differences in their abundance on the cell surface, in their interaction affinities for each other, and by differences in their relative position to each other within the plasma membrane.

## Conclusions

Taken together, our findings on heteromerization between α_1_-AR subtypes and on heteromerization between AVPR1A and α_1_-AR subtypes in hVSMCs in the present study confirm previous observations in recombinant expression systems and validate that findings on these recombinant receptors are applicable to their endogenously expressed counterparts. Furthermore, we demonstrate interdependency of the heteromerization patterns among six distinct endogenously expressed 7TM receptor protomers. Irrespective of the underlying molecular mechanisms, these findings suggest plasticity of 7TM receptor heteromerization in native cells and could be explained by a supramolecular organization of these receptors within dynamic clusters in the plasma membrane. The latter is consistent with our recent findings in recombinant systems and with the idea that such receptor hetero-oligomers form larger and possibly metastable signaling complexes [8, 9].

The present study is limited in scope because it was not designed to explore functional consequences associated with the observed re-arrangements of the receptor heteromers. Nevertheless, functional cross-talk between α_1_-ARs and AVPR1A is well documented [33–35] and we reported functional consequences of hetero-oligomerization between CXCR4, ACKR3, α_1_-ARs and AVPR1A on the molecular, cellular, and organ levels previously [9, 22–26]. Thus, it appears possible that the re-arrangements of the receptor heteromerization patterns in hVSMCs that we observed in the present study alter the pharmacological behavior of the individual receptor partners, which could contribute to the phenomenon of context-dependent GPCR signaling [36, 37].

In addition, our observations advise caution in the interpretation of functional consequences after GPCR knockdown in experimental models, because alterations of the heteromerization patterns among other receptor partners may alter physiological and pathological responses, in particular in more complex systems, such as studies on the function of isolated organs or in *in vivo* experiments.
